# Comparative transcriptome analysis of the main beam and brow tine of sika deer antler provides insights into the molecular control of rapid antler growth

**DOI:** 10.1186/s11658-020-00234-9

**Published:** 2020-09-07

**Authors:** Baojin Yao, Chaonan Wang, Zhenwei Zhou, Mei Zhang, Daqing Zhao, Xueyuan Bai, Xiangyang Leng

**Affiliations:** 1 Jilin Ginseng Academy, Changchun University of Chinese Medicine, Changchun, 130117 Jilin China; 2 College of traditional Chinese medicine, Changchun University of Chinese Medicine, Changchun, 130117 China; 3 Innovation Practice Center, Changchun University of Chinese Medicine, Changchun, 130117 Jilin China; 4 The Affiliated Hospital of Changchun University of Chinese Medicine, Changchun, 130117 Jilin China

**Keywords:** Deer antler, Main beam, Brow tine, RNA-Seq, Molecular mechanism

## Abstract

**Background:**

Deer antlers have become a valuable model for biomedical research due to the capacities of regeneration and rapid growth. However, the molecular mechanism of rapid antler growth remains to be elucidated. The aim of the present study was to compare and explore the molecular control exerted by the main beam and brow tine during rapid antler growth.

**Methods:**

The main beams and brow tines of sika deer antlers were collected from Chinese sika deer (*Cervus nippon*) at the rapid growth stage. Comparative transcriptome analysis was conducted using RNA-Seq technology. Differential expression was assessed using the DEGseq package. Functional Gene Ontology (GO) enrichment analysis was accomplished using a rigorous algorithm according to the GO Term Finder tool, and KEGG (Kyoto Encyclopedia of Genes and Genomes) pathway enrichment analysis was accomplished with the R function phyper, followed by the hypergeometric test and Bonferroni correction. Quantitative real-time polymerase chain reaction (qRT-PCR) was carried out to verify the RNA levels for differentially expressed mRNAs.

**Results:**

The expression levels of 16 differentially expressed genes (DEGs) involved in chondrogenesis and cartilage development were identified as significantly upregulated in the main beams, including transcription factor SOX-9 (Sox9), collagen alpha-1(II) chain (Col2a1), aggrecan core protein (Acan), etc. However, the expression levels of 17 DEGs involved in endochondral ossification and bone formation were identified as significantly upregulated in the brow tines, including collagen alpha-1(X) chain (Col10a1), osteopontin (Spp1) and bone sialoprotein 2 (Ibsp), etc.

**Conclusion:**

These results suggest that the antler main beam has stronger growth capacity involved in chondrogenesis and cartilage development compared to the brow tine during rapid antler growth, which is mainly achieved through regulation of Sox9 and its target genes, whereas the antler brow tine has stronger capacities of endochondral bone formation and resorption compared to the main beam during rapid antler growth, which is mainly achieved through the genes involved in regulating osteoblast and osteoclast activities. Thus, the current research has deeply expanded our understanding of the intrinsic molecular regulation displayed by the main beam and brow tine during rapid antler growth.

## Background

Deer antlers, which represent an extreme example of mammalian regeneration and rapid growth, have become a valuable model for biomedical research, including skeletal development and regeneration [1]. Typically, antlers grow with an S-shape growth curve. In other words, antlers grow slowly during the first 30–45 days, which usually occurs in spring. Then, antler growth enters a period of rapid exponential growth for 60–80 days, with a maximum growth rate up to 2 cm per day. Finally, antlers stop growing and gradually become completely ossified [2]. Antlers develop from the pedicles, which are permanent bony protuberances of the frontal skull [3]. When pedicles grow to 5–6 cm in height, the first antlers begin to generate spontaneously from the pedicle tips. The first antlers are usually small and unbranched spikes, whereas regenerated antlers, which refer to the antlers regenerated in the following years, successively increase in size and morphological complexity [4]. During the initial stages, the incipient antler forms a saddle-like structure, which consists of a main beam and a brow tine similar in length. As the antler grows, especially during the rapid growth stage, it will form a branched shape with a longer main beam and a shorter brow tine [5, 6]. At the completion of antler growth, the main beam contributes to more than 70% of the total antler volume [7].

In recent years, numerous studies have been conducted to analyze the gene expression patterns during antler growth. Ba and colleagues performed transcriptomic analysis of different tissue layers in the growth center of sika deer at about 30 days after casting the previous antler, and identified 370 hub genes which were mainly involved in mesenchymal progenitor cell proliferation, chondrogenesis, osteogenesis and angiogenesis [8]. Yao and colleagues performed transcriptomic analysis on the antler tips from sika deer to investigate underlying gene regulation in disparate growth layers in the fast growth phase (60 days after casting the previous antler), and discovered a string of genes that participate in the processes from the compression of mesenchymal cell to the differentiation of chondrocytes, namely chondrogenesis [9]. Another study, conducted using RNA-Seq technology by Yao and his colleagues, demonstrated that Sox9 functioned as a primary modulator during sika antler development by regulating multiple cell types from the mesenchymal precursor cells to the subsequently differentiated chondrocytes [10]. By analyzing the gene expression patterns of transcription factors, signaling molecules and extracellular matrices in the antler growth centers of sika deer at rapid growth and ossification stages, researchers also discovered a string of functional genes participating in the governance of rapid antler growth [11–13]. In addition, several studies were conducted by performing the analyses of RT-PCR, microarray and immunohistochemistry to identify the regulatory factors that controlled antler growth [14–20].

However, little is known regarding the differences of gene expression between main beam and brow tine during rapid antler growth. In the present study, we performed an RNA-Seq based transcriptome comparison in the main beams and brow tines of sika deer antler growth centers at the rapid growth stage. Our results suggest that the upregulated genes in the main beams are mainly involved in regulating chondrogenesis and cartilage development, whereas the upregulated genes in the brow tines are mainly involved in regulating endochondral ossification and bone formation.

## Methods

### Sample collection

The main beams and brow tines from two branched sika deer antlers (Fig. S1) were cut off at a deer farm in Changchun, China, from three Chinese sika deer (*Cervus nippon*) at the age of four in the fast growth phase (60 days after antlers had fallen off) under anesthesia. All the procedures of experimental operations complied with the Institutional Animal Care and Use Committee of Changchun University of Chinese Medicine (No. ccucm-2017-0015). The distal antler tips (growth centers) were collected as previously described [21]. All samples were chopped into 1-mm^3^ fragments and instantly immersed and preserved in liquid nitrogen after flushing with ice-cold distilled water.

### RNA isolation, library construction and sequencing

Total RNA was isolated using a TRIzol reagent (Invitrogen, USA) as stated by the company’s specification. RNA integrity was measured by a Bioanalyzer 2100 system (Agilent Technologies, USA), and only those with the value of RNA integrated number (RIN) ≥8 were picked up for the construction of the cDNA library with a TruSeq Stranded mRNA kit (Illumina, USA). Briefly, 200 ng of qualified total RNA was depurated by magnetic beads conjugated with oligo-dT (Life Technologies, USA) to generate polyadenylated mRNA. Following purification the mRNA was fragmented into small pieces. The mRNA fragments were random primed and reverse transcribed into double-stranded cDNAs, followed by RNA degradation by RNase, adenylation of 3′ end and paired-end sequencing adapter connection. The constructed libraries were further enriched by PCR amplification. A commercial Illumina HiSeq 2000 sequencer (Illumina, USA) was used to perform the subsequent sequencing.

### Transcriptome assembly and annotation

By the end of sequencing, raw reads generated with FASTQ format were processed to obtain clean reads by removing adapter sequences and low-quality reads (referred to reads that had a quality score (Q score) ≤ 20, equivalent to an error rate of ≥ 1% according to the process of base calling). De novo transcript assembly was accomplished using the Trinity program [22]. For annotation, the assembled transcripts were annotated by searching against the NCBI non-redundant (nr) and Swiss-Prot protein databases using the BLASTX program with a threshold of E-value ≤10^− 5^.

### Expression calculation and differentially expressed gene analysis

Gene expression level was calculated using a rigorous algorithm, namely FPKM (fragments per kilobase of transcript per million fragments mapped) [23]. Differential expression was assessed using a DEGseq R package [24]. Differentially expressed genes (DEGs) were defined by criteria of fold change ≥2 and false discovery rate (FDR) ≤0.01 [25].

### Functional and pathway enrichment analysis of DEGs

The enrichment analysis of functional Gene Ontology (GO) was accomplished by applying a rigorous algorithm according to the GO Term Finder tool, and the enrichment analysis of the Kyoto Encyclopedia of Genes and Genomes (KEGG) pathway was accomplished by applying a rigorous algorithm complied with the R function phyper, followed by the hypergeometric test and Bonferroni correction. After adjustment by multiple testing methods, only those with an adjusted *p*-value (namely Q-value) < 0.05 were defined as significantly enriched GO terms and pathways in the DEGs [26].

### Quantitative real-time PCR verification

RNA-seq data were further verified by quantitative real-time PCR (qRT-PCR). Briefly, RNA was isolated and purified as described above, and reverse transcription reaction was performed using an iScript cDNA Synthesis Kit (Bio-Rad, USA) as stated by the company’s specification. PCR conditions were set up using an SsoAdvanced Universal SYBR Green Supermix (Bio-Rad, USA) as stated by the company’s specification with specifically designed primers for each gene. PCR reactions were carried out on a CFX Connect Real-Time System (Bio-Rad, USA) under the following experimental conditions: 1 cycle for 30 s at 95 °C; 39 cycles for 10 s at 95 °C and 1 cycle for 30 s at 60 °C. The melt curves were generated by heating from 65 to 95 °C with a 0.5 °C increment. Relative mRNA levels were standardized to the internal reference gene, ribosomal protein L40 (Rpl40), and calculated with a rigorous algorithm according to the 2^-ΔΔCT^ method [10, 27]. Data are presented as the mean ± standard deviation of multiple independent experiments with each in technical triplicates.

## Results

### Transcriptome sequencing and de novo assembly

After Illumina transcriptome sequencing, 45,113,170 and 44,669,512 raw reads were obtained from the main beams and brow tines of the antler tips in the fast growth phase (60 days), respectively. After trimming off the adapter sequences and removing the low-quality reads, 40,479,562 (main beams) and 39,636,772 (brow tines) clean reads were obtained. The data sets were uploaded into the Sequence Read Archive (SRA) database under the accession number SRP114993, which is located in the National Centre of Biotechnology Information (NCBI). After de novo assembly, 74,707 (main beams) and 73,674 (brow tines) unigenes were obtained, with the average length of 516 and 488 nt, respectively. The detailed statistics of sequencing and read assembly are shown in Supplementary Table S1.

### Transcriptome annotation and differentially expressed gene analysis

Assembled sequences were subjected to the BLASTX program by searching against the NCBI non-redundant and Swiss-Prot protein databases using a threshold of E-value ≤10^− 5^. 36,813 genes returned a BLAST result under the threshold of E-value ≤10^− 5^, and 1093 genes were identified as differentially expressed genes (DEGs) according to the DEGseq analysis under the criteria of fold change ≥2 and a false discovery rate (FDR) ≤0.01. Among these DEGs, 778 genes were identified as highly expressed genes in the main beams, whereas 315 genes were identified as highly expressed genes in the brow tines. As shown in Supplementary Table S2, the top 30 highly expressed DEGs in the main beams mainly included fibronectin (Fn1), collagen alpha-1(II) chain (Col2a1), neuroblast differentiation-associated protein AHNAK (Ahnak), actin, cytoplasmic 2 (Actg1), matrix-remodeling-associated protein 5 (Mxra5), aggrecan core protein (Acan), ubiquitin carboxyl-terminal hydrolase isozyme L1 (Uchl1), transcription factor SOX-9 (Sox9), Septin-9 (Sept9) and cAMP-dependent protein kinase type I-alpha regulatory subunit (Prkar1a), etc. As shown in Supplementary Table S3, the top 30 highly expressed DEGs in the brow tines mainly included NADH-ubiquinone oxidoreductase chain 1 (Mtnd1), collagen alpha-1(X) chain (Col10a1), osteopontin (Spp1), bone sialoprotein 2 (Ibsp), tartrate-resistant acid phosphatase type 5 (Acp5), osteocalcin (Ocn), protein S100-A9 (S100a9), ethylmalonyl-CoA decarboxylase (Echdc1), periostin (Postn) and matrix metalloproteinase-9 (Mmp9), etc.

### GO and KEGG enrichment analysis

**Fig. 1:**
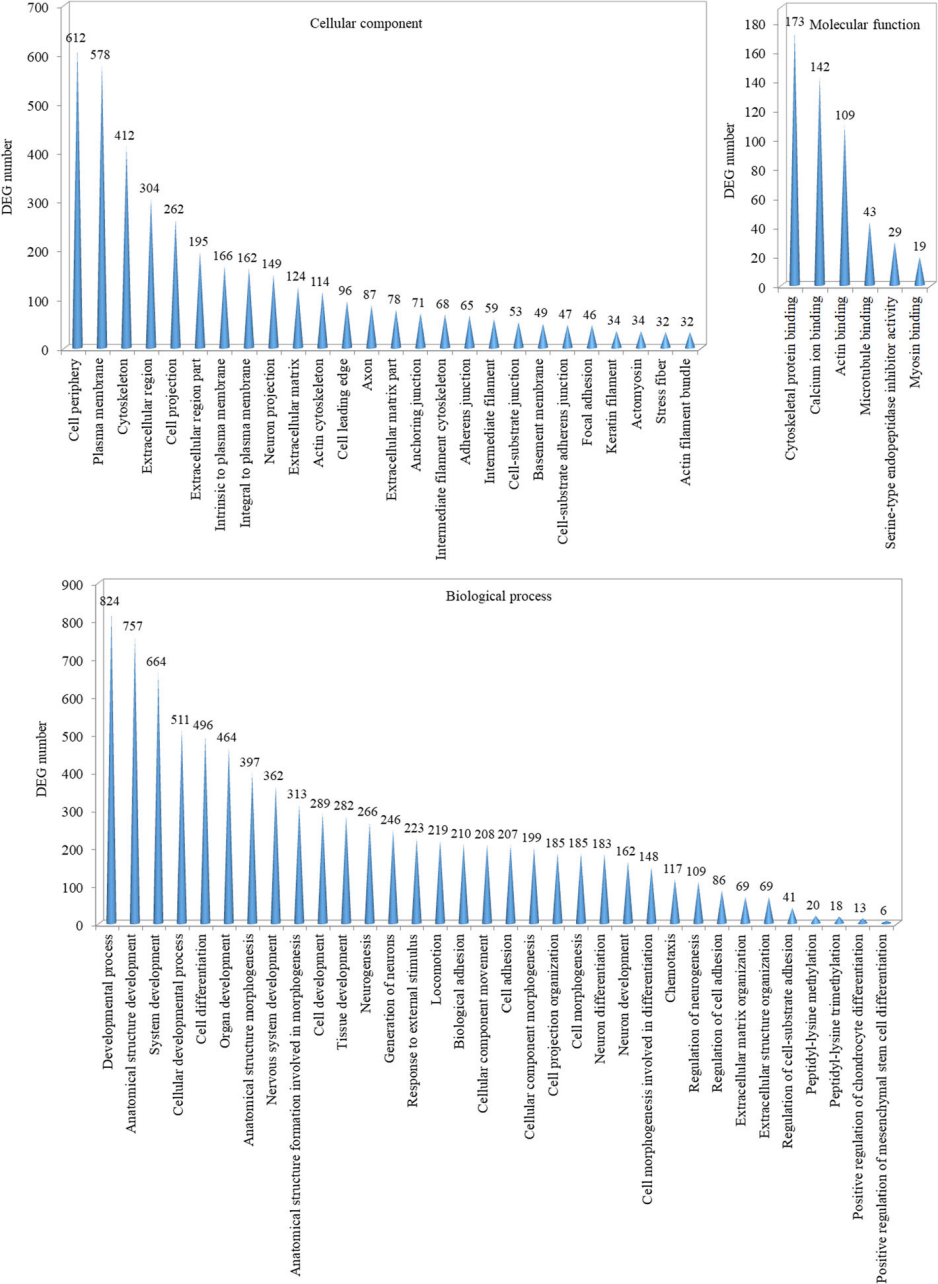
Histogram presentation of enriched GO classifications of DEGs. The data were classified into the following three main categories: cellular component, molecular function and biological process. The ordinate indicates the number of DEGs matched up with the GO term, while the abscissa indicates the exact name of each GO term

As shown in Fig. 1, the classification of cellular components indicated that the majority of the DEGs were situated in the cell periphery, plasma membrane, cytoskeleton, extracellular region and cell projection. The classification of molecular function indicated that the main functions of these DEGs were cytoskeletal protein binding, calcium ion binding and actin binding. The classification of biological processes indicated that these DEGs were mainly involved in developmental processes, anatomical structure development, system development, cellular developmental processes and cell differentiation.

**Fig. 2:**
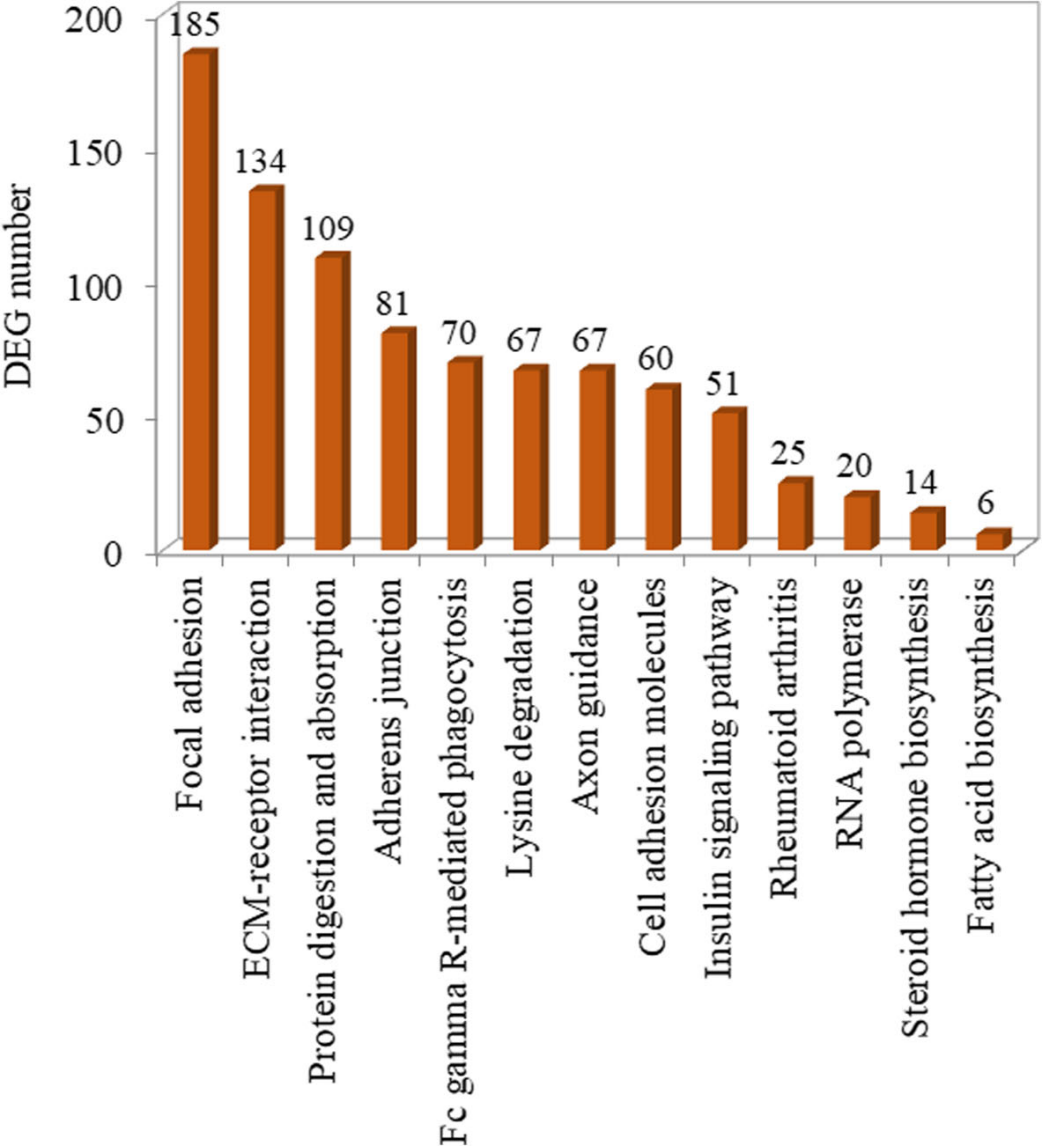
Histogram presentation of enriched KEGG pathways of DEGs. The ordinate indicates the number of DEGs matched up with the enriched pathway, while the abscissa indicates the exact name of each pathway

To explore the possible physiological processes and pathways of the DEGs, we further mapped these DEGs to the KEGG database. As shown in Fig. 2, the DEGs mainly joined in the pathways including focal adhesion, ECM-receptor interaction, protein digestion and absorption, adherens junction, Fc gamma R-mediated phagocytosis, lysine degradation, axon guidance, cell adhesion molecules and insulin signaling pathway, etc.

### Identification of DEGs involved in chondrogenesis, cartilage development, endochondral ossification and bone formation

Since antler growth is driven by the antler tip, a place considered to be the growth center, through processes that include chondrogenesis, cartilage development, endochondral ossification and bone formation [10], we further screened and identified DEGs that were involved in the above processes. As shown in Table 1, the expression levels of 16 DEGs involved in chondrogenesis and cartilage development were identified as significantly upregulated in the main beams, including fibronectin (Fn1), collagen alpha-1(II) chain (Col2a1), aggrecan core protein (Acan), transcription factor SOX-9 (Sox9), basement membrane-specific heparan sulfate proteoglycan core protein (Hspg2) and versican core protein (Vcan), etc. However, as shown in Table 2, the expression levels of 17 DEGs involved in endochondral ossification and bone formation were identified as significantly upregulated in the brow tines, including collagen alpha-1(X) chain (Col10a1), osteopontin (Spp1), tartrate-resistant acid phosphatase type 5 (Acp5), bone sialoprotein 2 (Ibsp), osteocalcin (Ocn) and protein S100-A9 (S100a9), etc.

**Table 1:** Identification of DEGs involved in chondrogenesis and cartilage development

Gene name	Gene expression level (FPKM)		Fold change	FDR
	Brow tines	Main beams	log_2_ FPKM (main beams/brow tines)	
Fibronectin (Fn1)	44.60	190.37	2.09	0
Collagen alpha-1(II) chain (Col2a1)	20.41	101.11	2.31	0
Aggrecan core protein (Acan)	7.71	31.83	2.05	4.02E-252
Transcription factor SOX-9 (Sox9)	5.83	24.54	2.07	1.59E-52
Basement membrane-specific heparan sulfate proteoglycan core protein (Hspg2)	3.75	18.24	2.28	0
Versican core protein (Vcan)	1.40	6.78	2.28	5.44E-22
Pappalysin-2 (Pappa2)	1.40	6.53	2.22	8.90E-19
Protein sidekick-2 (Sdk2)	1.30	5.84	2.17	2.22E-30
Neurofibromin (Nf1)	0.95	5.73	2.59	1.99E-06
Transcription factor SOX-6 (Sox6)	1.07	4.50	2.07	1.38E-14
Runt-related transcription factor 2 (Runx2)	0.40	3.82	3.26	2.97E-07
Forkhead box protein L2 (Foxl2)	0.00	3.57	11.80	2.64E-11
cGMP-dependent protein kinase 2 (Prkg2)	0.33	2.92	3.14	5.17E-08
Protein FAM101B (Fam101b)	0.06	1.81	4.84	4.08E-07
Collagen alpha-1(XI) chain (Col11a1)	0.12	1.66	3.79	1.38E-63
Fibroblast growth factor receptor 3 (Fgfr3)	0.00	0.18	7.47	1.82E-04

**Table 2:** Identification of DEGs involved in endochondral ossification and bone formation

Gene name	Gene expression level (FPKM)		Fold change	FDR
	Brow tines	Main beams	log_2_ FPKM (main beams/brow tines)	
Collagen alpha-1(X) chain (Col10a1)	1008.17	231.43	−2.12	0
Osteopontin (Spp1)	743.90	112.04	−2.73	0
Bone sialoprotein 2 (Ibsp)	495.27	112.68	−2.14	0
Tartrate-resistant acid phosphatase type 5 (Acp5)	316.17	22.63	−3.80	0
Osteocalcin (Ocn)	123.45	28.47	−2.12	3.24E-182
Protein S100-A9 (S100a9)	95.16	15.14	−2.65	8.41E-284
Periostin (Postn)	34.67	3.13	−3.47	3.76E-222
Matrix metalloproteinase-9 (Mmp9)	24.65	4.94	−2.32	1.43E-206
Ras-related C3 botulinum toxin substrate 2(Rac2)	17.91	3.87	−2.21	1.53E-136
C-type lectin domain family 3 member A (Clec3a)	17.57	2.49	−2.82	1.21E-148
Metalloendopeptidase homolog PEX (Phex)	12.26	1.37	−3.16	2.49E-43
Dentin matrix acidic phosphoprotein 1 (Dmp1)	10.88	0.10	−6.80	6.58E-185
Sorting nexin-10 (Snx10)	10.70	2.27	−2.23	5.56E-45
Cytokine-like protein 1 (Cytl1)	8.39	1.63	−2.36	5.85E-30
Megakaryocyte-associated tyrosine-protein kinase (Matk)	7.78	0.88	−3.14	9.94E-23
Disintegrin and metalloproteinase domain-containing protein 8 (Adam8)	5.51	1.17	−2.24	7.38E-17
Osteoclast-associated immunoglobulin-like receptor (Oscar)	4.21	0.61	−2.80	6.95E-12

### qRT-PCR validation

**Fig. 3:**
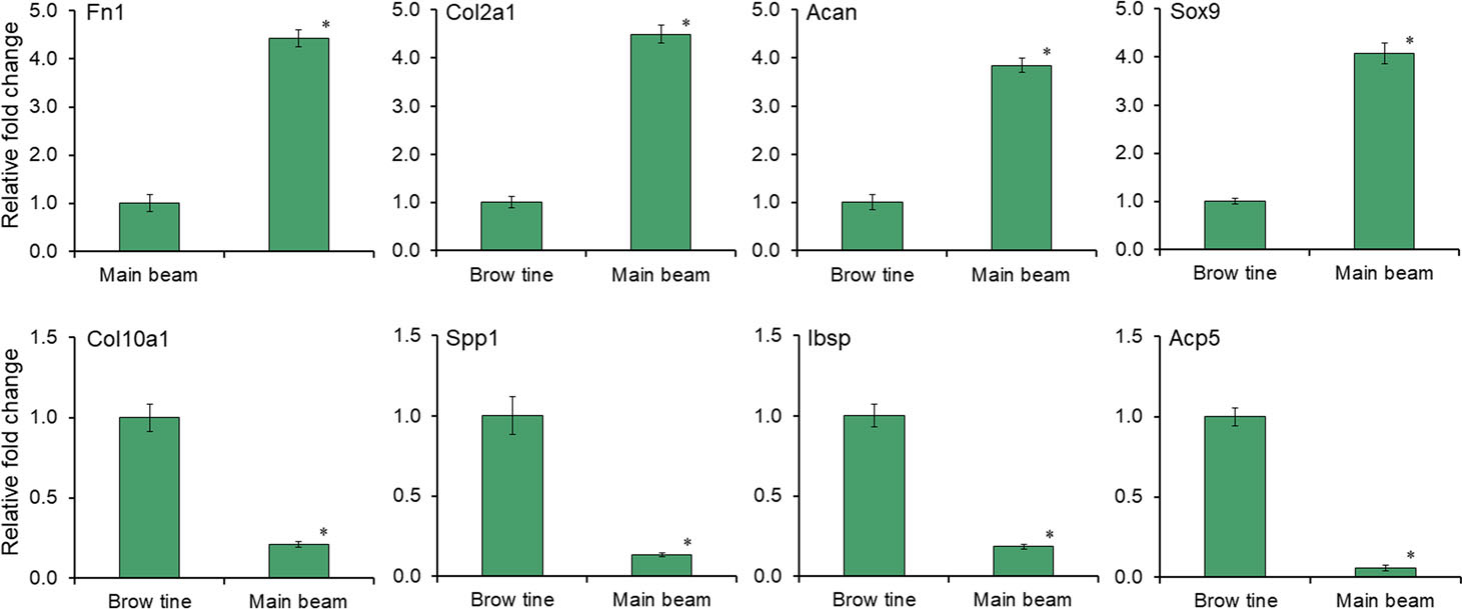
The relative gene expression levels of DEG verified by qRT-PCR. Relative mRNA levels were standardized to the internal reference gene, ribosomal protein L40 (Rpl40), and calculated with a rigorous algorithm according to the 2^-ΔΔCT^ method. The quantitative change of each gene that was represented by relative fold-change in the main beams was normalized and compared to the brow tines, separately. Data are presented as the mean ± standard deviation of multiple independent experiments with each in technical triplicates. * indicates that the *p* value is less than 0.001 in the t-test

To validate the RNA-Seq results, we selected 8 DEGs that were involved in chondrogenesis, cartilage development, endochondral ossification and bone formation, i.e. Fn1, Col2a1, Acan, Sox9, Col10a1, Spp1, Ibsp and Acp5, and investigated their expression levels by qRT-PCR assay. Primers were designed using the assembled sequences from RNA-Seq analysis, as shown in Supplementary Table S4. The quantitative change of each gene that was represented by relative fold-change in the main beams was normalized and compared to the brow tines, separately. As shown in Fig. 3, the result of qRT-PCR was similar to that observed by RNA-Seq.

## Discussion

Deer antlers are distinctive skull appendages with capacities of cyclic regeneration and fast growth. Thus, they have been considered as valuable models for studying organ growth and regeneration. However, the distinct molecular mechanism that drives main beam and brow tine growth during rapid antler growth is still largely unknown. In the current study, we used cutting-edge RNA-Seq technology to obtain novel insights into the expression levels, functions and pathways of the differentially expressed genes (DEGs) involved in main beam and brow tine growth during rapid antler growth.

Our analysis revealed that the most highly expressed DEGs in the main beams mainly consisted of genes involved in regulating chondrogenesis and cartilage development, such as Fn1, Col2a1, Acan and Sox9, whereas the most highly expressed DEGs in the brow tines mainly consisted of genes involved in regulating endochondral ossification and bone formation, such as Col10a1, Spp1, Ibsp, Acp5, Ocn, Postn and Mmp9. These results suggest that the growth pattern of the antler main beam is quite different compared to the growth pattern of the brow tine during rapid antler growth. In line with our findings, Li and colleagues demonstrated that the antler tips of the main beam and brow tine were self-formed at the dorsal and ventral corners of the pedicles that generate the antler blastemata. Histological analysis showed that the three major zones comprising the mesenchymal zone, precartilaginous zone, and transitional zone in the growth center of the main beam are much thicker than those of the growth center of brow tine [28].

According to the GO functional and KEGG pathway enrichment analyses of DEGs, a majority of these DEGs were located in the regions of the cell periphery, plasma membrane, cytoskeleton and extracellular region with molecular functions involved in cytoskeletal protein binding, calcium ion binding and actin binding, and mainly involved in the processes of development, anatomical structure development, system development, cellular development and cell differentiation. According to the KEGG pathway enrichment analyses of DEGs, a majority of these DEGs mainly participated in the pathways including focal adhesion, ECM-receptor interaction, protein digestion and absorption and adherens junction. Thus, these results further confirm that antler main beam growth indeed differs from brow tine growth during rapid antler growth due to the changes involved in cytoskeleton structure, ECM interaction and signaling transduction.

In order to further delineate the difference between the main beam and brow tine, and explore the underlying molecular mechanism involved in antler main beam and brow tine growth during rapid antler growth, we screened and analyzed the expression levels of differentially expressed genes related to chondrogenesis, cartilage development, endochondral ossification and bone formation. We identified 16 DEGs that were involved in chondrogenesis and cartilage development and were significantly upregulated in the main beams, i.e. Fn1, Col2a1, Acan, Sox9, Hspg2, Vcan, Pappa2, Sdk2, Nf1, Sox6, Runx2, Foxl2, Prkg2, Fam101b, Col11a1 and Fgfr3. Fn1 and Vcan are essential matrix proteins for cell condensation during chondrogenesis [29]. Sox9 serves as a master transcription factor involved in multiple chondrocyte differentiation pathways [30]. Sox9 was needed for the expression of several genes including Col2a1, Col11a1, Acan and Sox6 during cartilage development [31]. Hspg2 is an essential proteoglycan that is deposited within the pericellular matrix surrounding chondrocytes, and is indispensable for the development of articular cartilage [32]. Pappa2 is a huge and essential metalloproteinase that is required for normal development of cranial cartilage [33]. Sdk2 is an adhesion molecule that is very selectively expressed in fetal cartilage [34]. Nf1 is a cytoplasmic protein that is predominantly expressed in the maturing and hypertrophic cartilage during fracture healing [35]. Runx2 is an essential transcription factor that regulates endochondral ossification through controlling both chondrocyte proliferation and differentiation [36]. Foxl2 is a critical regulator that plays pivotal roles in regulating postnatal growth and embryonic bone and cartilage formation [37]. Prkg2 is a kinase of protein that plays a pivotal role in converting proliferating chondrocytes into hypertrophic chondrocytes through endochondral ossification in the growth plate [38]. Fam101b is an actin binding protein predominantly expressed in developing cartilage, which plays a crucial role in regulating chondrocyte proliferation and differentiation [39]. Fgfr3 acts as a cell-surface receptor for fibroblast growth factors and is mainly expressed in the limb chondrocytes at an early development stage and later in the growth plate chondrocytes in the reserve and proliferating regions [40]. These results suggest that the antler main beam has stronger growth capacity involved in chondrogenesis and cartilage development compared to the brow tine during rapid antler growth, which is mainly achieved through the regulation of Sox9 and its downstream target genes.

Consistently with the above findings, compared with the main beams, 17 DEGs involved in endochondral ossification and bone formation were identified as significantly upregulated in the brow tines: Col10a1, Spp1, Ibsp, Acp5, Ocn, S100a9, Postn, Mmp9, Rac2, Clec3a, Phex, Dmp1, Snx10, Cytl1, Matk, Adam8 and Oscar. Co10a1, a specific marker of hypertrophic chondrocytes, plays a crucial role during endochondral bone formation [41]. Spp1, Ibsp, Acp5, Ocn, Postn and Mmp9 are genes associated with bone matrix formation and turnover [42]. S100a9 is a calcium-binding protein that is involved in cartilage matrix calcification, bone formation and resorption [43]. Rac2 is a member of the Rac subfamily of Rho GTPases, which is required for optimal osteoclast differentiation during osteoclastogenesis [44]. Clec3a is a member of the superfamily of C-type lectins, and is involved in bone formation [45]. Phex is a phosphate-regulating gene that plays a critical role in regulating osteoblast mineralization [46]. Dmp1, an acidic noncollagenous phosphoprotein, is critical for osteoblast differentiation and mineralization of the bone extracellular matrix [47]. Snx10, a member of the sorting nexin family of proteins, is expressed in osteoclasts and is required for osteoclast activity [48]. Cytl1 serves as a secreted cytokine candidate that positively modulates bone mass through regulating osteoclastogenesis [49]. Matk is a c-Src tyrosine kinase that is essential for modulating osteoclast and osteoblast activities involved in bone metabolism [50]. Adam8 is an autocrine/paracrine metalloproteinase highly expressed in osteoclast precursors, and significantly stimulates osteoclastogenesis [51]. Oscar is a crucial osteoimmunological mediator that serves as a costimulatory molecule for osteoclast differentiation [52]. These results suggest that the antler brow tine has stronger capacities of endochondral bone formation and resorption compared to the main beam during rapid antler growth, which is mainly achieved through regulating osteoblast and osteoclast activities.

## Conclusion

In the present study, we demonstrated that antler main beam growth indeed differs from brow tine growth during rapid antler growth. The antler main beam has stronger growth capacity in chondrogenesis and cartilage development compared to the brow tine during rapid antler growth, which is mainly achieved through the regulation of Sox9 and its downstream target genes, such as Col2a1, Acan, Sox9, Sox6, Col11a1 and Fgfr3. However, the antler brow tine has stronger capacities of endochondral bone formation and resorption compared to the main beam during rapid antler growth, which is mainly achieved through the regulation of osteoblast and osteoclast activities by upregulated genes, such as Col10a1, Spp1, Ibsp, Acp5, Ocn, S100a9, Postn, Mmp9, Rac2, Clec3a, Phex, Dmp1, Snx10, Cytl1, Matk, Adam8 and Oscar. Thus, the current research has deeply expanded our understanding of the intrinsic molecular regulation displayed by the main beam and brow tine during rapid antler growth.

## Supplementary information

**Additional file 1: Table S1.** Statistical summary of sequencing and read assembly

**Additional file 2: Table S2.** List of the top 30 highly expressed DEGs in the main beams (main beams vs. brow tines)

**Additional file 3: Table S3.** List of the top 30 highly expressed DEGs in the brow tines (main beams vs. brow tines)

**Additional file 4: Table S4.** Gene-specific primers used for qRT-PCR verification

**Additional file 5: Figure S1.** Schematic diagram of antler sampling. The picture shows representative two-branched antlers with main beams (A and B) and brow tines (C and D), and the red square dotted lines indicate the sampling regions (the distal 5 cm of the antler tips).

## Data Availability

The data from this study are available from the author for correspondence on reasonable request.

## Abbreviations

RNA-Seq: RNA sequencing
DEGs: Differentially expressed genes
FDR: False discovery rate
qRT-PCR: Quantitative real-time PCR
FPKM: Fragments per kilobase of transcript per million fragments mapped
GO: Gene Ontology
KEGG: Kyoto Encyclopedia of Genes and Genomes
SRA: Sequence Read Archive
NCBI: National Centre of Biotechnology Information
