# Supplementary material for: Comparative transcriptome analysis of the main beam and brow tine of sika deer antler provides insights into the molecular control of rapid antler growth
Source: Cell Mol Biol Lett. 2020 Sep 7;25:42. doi: 10.1186/s11658-020-00234-9 (PMC7487962; doi:10.1186/s11658-020-00234-9)
Supplement: Supplementary file 1 — Additional file 1: Table S1. Statistical summary of sequencing and read assembly [file 11658_2020_234_MOESM1_ESM.doc]

Table S1 Statistical summary of sequencing and read assembly

| Statistics | Main beams | Brow tines |
| --- | --- | --- |
| Raw reads | 45,113,170 | 44,669,512 |
| Clean reads | 40,479,562 | 39,636,772 |
| Q30 percentage | 96.67 | 96.25 |
| N percentage | 0.00 | 0.00 |
| GC percentage | 51.75 | 49.80 |
| Unigenes | 74,707 | 73,674 |
| Unigene average length (nt) | 516 | 488 |
