# Supplementary material for: Comparative transcriptome analysis of the main beam and brow tine of sika deer antler provides insights into the molecular control of rapid antler growth
Source: Cell Mol Biol Lett. 2020 Sep 7;25:42. doi: 10.1186/s11658-020-00234-9 (PMC7487962; doi:10.1186/s11658-020-00234-9)
Supplement: Supplementary file 2 — Additional file 2: Table S2. List of the top 30 highly expressed DEGs in the main beams (main beams vs. brow tines) [file 11658_2020_234_MOESM2_ESM.doc]

Table S2 List of the top 30 highly expressed DEGs in the main beams (main beams vs. brow tines)

| Gene name | Gene expression level (FPKM) | | Fold change | FDR |
| --- | --- | --- | --- | --- |
|  | Brow tines | Main beams | log_2_ FPKM (main beams/brow tines) |  |
| Fibronectin (Fn1) | 44.60 | 190.37 | 2.09 | 0 |
| Collagen alpha-1(II) chain (Col2a1) | 20.41 | 101.11 | 2.31 | 0 |
| Neuroblast differentiation-associated protein AHNAK (Ahnak) | 14.75 | 97.52 | 2.73 | 5.36E-161 |
| Actin, cytoplasmic 2 (Actg1) | 16.01 | 88.62 | 2.47 | 0 |
| Matrix-remodeling-associated protein 5 (Mxra5) | 9.85 | 50.07 | 2.35 | 0 |
| Aggrecan core protein (Acan) | 7.71 | 31.83 | 2.05 | 4.02E-252 |
| Ubiquitin carboxyl-terminal hydrolase isozyme L1 (Uchl1) | 7.13 | 28.92 | 2.02 | 2.08E-92 |
| Transcription factor SOX-9 (Sox9) | 5.83 | 24.54 | 2.07 | 1.59E-52 |
| Septin-9 (Sept9) | 2.49 | 20.17 | 3.02 | 3.61E-116 |
| cAMP-dependent protein kinase type I-alpha regulatory subunit (Prkar1a) | 3.03 | 19.33 | 2.67 | 7.48E-55 |
| Myosin light chain 1/3, skeletal muscle isoform (Myl1) | 0.49 | 19.03 | 5.29 | 4.75E-111 |
| Liprin-alpha-1 (Ppfia1) | 3.99 | 18.44 | 2.21 | 8.70E-42 |
| Basement membrane-specific heparan sulfate proteoglycan core protein (Hspg2) | 3.75 | 18.24 | 2.28 | 0 |
| Baculoviral IAP repeat-containing protein 5 (Birc5) | 1.64 | 15.27 | 3.22 | 2.26E-25 |
| 39S ribosomal protein L36, mitochondrial (Mrpl36) | 2.24 | 12.98 | 2.54 | 2.81E-41 |
| Cytoplasmic dynein 1 heavy chain 1 (Dync1h1) | 2.56 | 12.65 | 2.30 | 2.83E-78 |
| Tyrosine-protein phosphatase non-receptor type 14 (Ptpn14) | 2.45 | 12.64 | 2.37 | 2.70E-54 |
| Splicing factor 3A subunit 2 (Sf3a2) | 2.73 | 11.26 | 2.04 | 1.57E-07 |
| Vacuolar protein sorting-associated protein 13B (Vps13b) | 2.40 | 11.18 | 2.22 | 6.98E-10 |
| Protocadherin Fat 1 (Fat1) | 1.63 | 11.06 | 2.76 | 7.27E-74 |
| Nuclear cap-binding protein subunit 1 (Ncbp1) | 2.12 | 10.64 | 2.33 | 4.52E-13 |
| Ras-associated and pleckstrin homology domains-containing protein 1 (Raph1) | 2.54 | 10.29 | 2.02 | 1.77E-21 |
| Nuclear receptor coactivator 2 (Ncoa2) | 1.88 | 10.16 | 2.43 | 1.34E-12 |
| Protein FAM178A (Fam178a) | 1.96 | 9.67 | 2.30 | 8.12E-08 |
| Probable ubiquitin carboxyl-terminal hydrolase FAF-X (Usp9x) | 1.60 | 9.63 | 2.59 | 8.19E-17 |
| Rho GTPase-activating protein 32 (Arhgap32) | 1.86 | 9.27 | 2.32 | 3.54E-09 |
| Antigen KI-67 (Mki67) | 2.29 | 9.20 | 2.00 | 5.69E-47 |
| Eukaryotic translation initiation factor 4 gamma 1 (Eif4g1) | 0.24 | 9.04 | 5.22 | 6.29E-19 |
| Homeodomain-interacting protein kinase 1 (Hipk1) | 1.91 | 8.70 | 2.19 | 5.35E-31 |
| Ceramide synthase 6 (Cers6) | 1.13 | 8.31 | 2.87 | 2.02E-14 |
