# Supplementary material for: Comparative transcriptome analysis of the main beam and brow tine of sika deer antler provides insights into the molecular control of rapid antler growth
Source: Cell Mol Biol Lett. 2020 Sep 7;25:42. doi: 10.1186/s11658-020-00234-9 (PMC7487962; doi:10.1186/s11658-020-00234-9)
Supplement: Supplementary file 3 — Additional file 3: Table S3. List of the top 30 highly expressed DEGs in the brow tines (main beams vs. brow tines) [file 11658_2020_234_MOESM3_ESM.doc]

Table S3 List of the top 30 highly expressed DEGs in the brow tines (main beams vs. brow tines)

| Gene name | Gene expression level (FPKM) | | Fold change | FDR |
| --- | --- | --- | --- | --- |
|  | Brow tines | Main beams | log_2_ FPKM (main beams/brow tines) |  |
| NADH-ubiquinone oxidoreductase chain 1 (Mtnd1) | 4459.45 | 886.59 | -2.33 | 0 |
| Collagen alpha-1(X) chain (Col10a1) | 1008.17 | 231.43 | -2.12 | 0 |
| Osteopontin (Spp1) | 743.90 | 112.04 | -2.73 | 0 |
| Bone sialoprotein 2 (Ibsp) | 495.27 | 112.68 | -2.14 | 0 |
| Tartrate-resistant acid phosphatase type 5 (Acp5) | 316.17 | 22.63 | -3.80 | 0 |
| Osteocalcin (Ocn) | 123.45 | 28.47 | -2.12 | 3.24E-182 |
| Protein S100-A9 (S100a9) | 95.16 | 15.14 | -2.65 | 8.41E-284 |
| Ethylmalonyl-CoA decarboxylase (Echdc1) | 60.87 | 7.86 | -2.95 | 0 |
| Periostin (Postn) | 34.67 | 3.13 | -3.47 | 3.76E-222 |
| Matrix metalloproteinase-9 (Mmp9) | 24.65 | 4.94 | -2.32 | 1.43E-206 |
| Late cornified envelope protein 1F (Lce1f) | 21.79 | 4.68 | -2.22 | 8.49E-71 |
| Ras-related C3 botulinum toxin substrate 2(Rac2) | 17.91 | 3.87 | -2.21 | 1.53E-136 |
| Keratin, type I cytoskeletal 10 (Krt10) | 17.63 | 0.65 | -4.77 | 4.35E-195 |
| C-type lectin domain family 3 member A (Clec3a) | 17.57 | 2.49 | -2.82 | 1.21E-148 |
| Kallikrein-7 (Klk7) | 16.75 | 1.90 | -3.14 | 1.18E-107 |
| Antimicrobial peptide NK-lysin (Nkl) | 12.67 | 1.38 | -3.20 | 8.07E-42 |
| Ras-related protein Rab-38 (Rab38) | 12.32 | 2.98 | -2.05 | 6.21E-46 |
| Metalloendopeptidase homolog PEX (Phex) | 12.26 | 1.37 | -3.16 | 2.49E-43 |
| High mobility group nucleosome-binding domain-containing protein 5 (Hmgn5) | 11.89 | 2.76 | -2.11 | 6.96E-47 |
| Late cornified envelope protein 5A (Lce5a) | 11.66 | 2.58 | -2.18 | 7.95E-23 |
| Group 10 secretory phospholipase A2 (Pla2g10) | 11.56 | 0.71 | -4.02 | 5.01E-38 |
| Luc7-like protein 3 (Luc7l3) | 11.48 | 1.12 | -3.36 | 3.77E-43 |
| Peptidoglycan recognition protein 1 (Pglyrp1) | 11.46 | 2.15 | -2.41 | 8.69E-34 |
| Lymphocyte activation gene 3 protein (Lag3) | 11.40 | 0.87 | -3.71 | 9.90E-16 |
| Kallikrein-9 (Klk9) | 11.32 | 0.99 | -3.51 | 3.96E-63 |
| Dentin matrix acidic phosphoprotein 1 (Dmp1) | 10.88 | 0.10 | -6.80 | 6.58E-185 |
| Sorting nexin-10 (Snx10) | 10.70 | 2.27 | -2.23 | 5.56E-45 |
| Phosphatidylethanolamine-binding protein 4 (Pebp4) | 10.53 | 1.25 | -3.07 | 4.17E-41 |
| Kinesin-like protein KIF20B (Kif20b) | 9.74 | 2.37 | -2.04 | 1.71E-41 |
| F-actin-capping protein subunit beta (Capzb) | 9.65 | 1.66 | -2.54 | 6.52E-64 |
