# Supplementary material for: Comparative transcriptome analysis of the main beam and brow tine of sika deer antler provides insights into the molecular control of rapid antler growth
Source: Cell Mol Biol Lett. 2020 Sep 7;25:42. doi: 10.1186/s11658-020-00234-9 (PMC7487962; doi:10.1186/s11658-020-00234-9)
Supplement: Supplementary file 4 — Additional file 4: Table S4. Gene-specific primers used for qRT-PCR verification [file 11658_2020_234_MOESM4_ESM.doc]

Table S4 Gene-specific primers used for qRT-PCR verification

| Gene | Primer | Sequence |
| --- | --- | --- |
| Fn1 | Forward primer | AGAAGTGAGTCCAGAGCAAAG |
|  | Reverse primer | CCCGAGAGTAATATTTCCAGCC |
| Col2a1 | Forward primer | GGAATTCGGTGTGGACATAGG |
|  | Reverse primer | CAGGTCAGATCAGCCATTCAG |
| Acan | Forward primer | TCTACCTCTACCCCAACCAG |
|  | Reverse primer | TGAAACACCTCGGAAGCAG |
| Sox9 | Forward primer | TGTTAGGCAGAGGTCCTCAC |
|  | Reverse primer | GTGGCCTTGAGGAAACTTGG |
| Col10a1 | Forward primer | GAGCGATACCAAACACCTAC |
|  | Reverse primer | CTTAGCGACACACCTTTACC |
| Spp1 | Forward primer | ACAACAAACAAAATACCCTCCC |
|  | Reverse primer | TCCATAAGCCACACTATCACC |
| Ibsp | Forward primer | GAAGAGGAGGAGGAAGAAGAG |
|  | Reverse primer | AGTGTGGTAGTGGAAAGGG |
| Acp5 | Forward primer | AGCATCACTTACATTGAAGCC |
|  | Reverse primer | CACAAGTTCCTCATCTGCC |
| Rpl40 | Forward primer | CCTTTCGTTGACTGGAGCAG |
|  | Reverse primer | CACGCTTGGGTATGTCCTTG |
