# Supplementary figures and images for: Comparative transcriptome analysis of the main beam and brow tine of sika deer antler provides insights into the molecular control of rapid antler growth
Source: Cell Mol Biol Lett. 2020 Sep 7;25:42. doi: 10.1186/s11658-020-00234-9 (PMC7487962; doi:10.1186/s11658-020-00234-9)

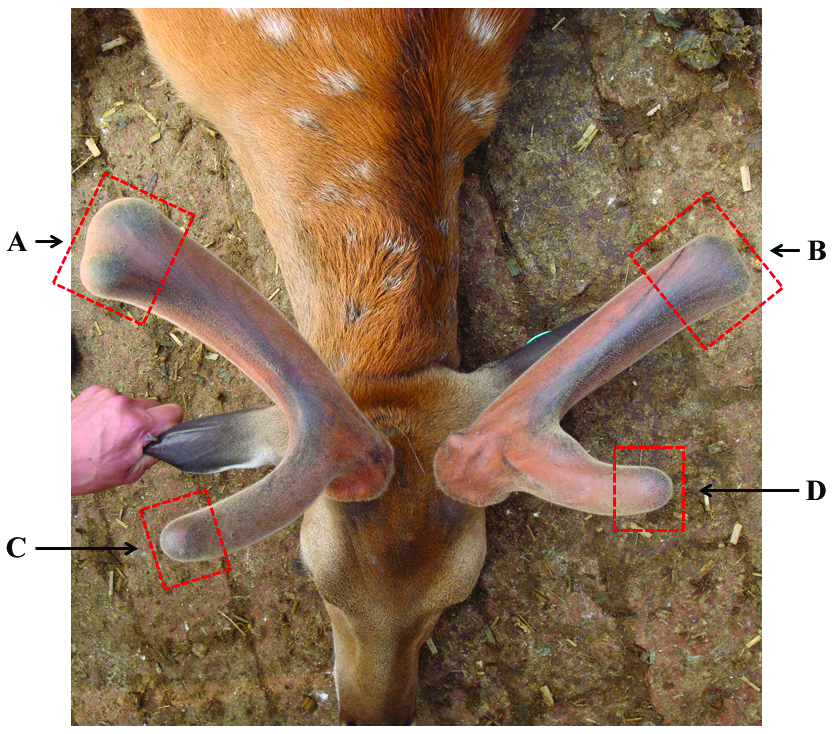

Supplement: Supplementary file 5 — Additional file 5: Figure S1. Schematic diagram of antler sampling. The picture shows representative two-branched antlers with main beams (A and B) and brow tines (C and D), and the red square dotted lines indicate the sampling regions (the distal 5 cm of the antler tips). [file 11658_2020_234_MOESM5_ESM.tif]
